# Efficacy of glucagon-like peptide 1 analogues in psoriasis: A cohort study

**DOI:** 10.1016/j.jdin.2026.04.004

**Published:** 2026-04-13

**Authors:** Danielle Bar, Felix Pavlotsky, Sharon Baum, Aviv Barzilai

**Affiliations:** a Department of Dermatology, Gray Faculty of Medical and Health Sciences, Tel-Aviv University, Tel-Aviv, Israel; b Division of Dermatology, Sheba Medical Center, Ramat-Gan, Israel; c Dermatopathology Service, Department of Pathology, Sheba Medical Center, Ramat Gan, Israel

**Keywords:** epidemiology, GLP-1 receptor agonist, immunometabolism, obesity, psoriasis, weight loss

## Abstract

**Background:**

Obesity-related metabolic inflammation contributes to psoriasis pathogenesis and is associated with reduced therapeutic response.

**Objectives:**

To investigate whether treatment with glucagon-like peptide-1 (GLP-1) analogs improves skin disease in patients who have obesity with psoriasis.

**Methods:**

Consecutive patients with psoriasis eligible for GLP-1 analog therapy (body mass index ≥27) were enrolled. A comparator cohort comprised patients who have obesity with psoriasis who did not receive GLP-1 therapy. The primary outcomes were the proportion of patients achieving an investigator's global assessment score of 0/1, and the correlation between weight reduction and improvement in skin disease.

**Results:**

An investigator's global assessment 0/1 response was achieved by 16 of 36 patients (44.4%) in the GLP-1 group compared with 7 of 58 patients (12.1%) in the control group (*P* < .001). Greater weight loss correlated with a more rapid response (τ = −0.59; *P* < .001). Among patients receiving biologic therapy, the addition of GLP-1 treatment was associated with superior response rates (*P* = .029).

**Conclusion:**

GLP-1 receptor agonists demonstrated efficacy in patients who have obesity with psoriasis and may represent a therapeutic alternative for individuals in whom conventional systemic or biologic therapies are contraindicated, or in those who prefer a non-immunomodulatory approach.

**Capsule Summary**

• Psoriasis is strongly associated with obesity and the metabolic syndrome; however, the therapeutic efficacy of GLP1 analogs has not been explored.
• This study demonstrates significant improvement in psoriatic skin disease among patients receiving GLP1 analogs.

## Introduction

Psoriasis is a chronic inflammatory skin disease affecting 3% of the general population.^1^ The systemic inflammation inherent to the disease predisposes patients to obesity and metabolic comorbidities, which parallel disease severity.^2^ Obesity, in turn, is not only an independent risk factor for psoriasis but also a well-recognized determinant of diminished therapeutic response to biologic agents.^3^^,^^4^ In the context of the escalating obesity epidemic, the intersection of obesity and psoriasis poses a therapeutic challenge.

Glucagon-like peptide-1 (GLP-1) receptor agonists have emerged as transformative agents in obesity management, producing sustained weight reductions of 15% to 20% while improving glycemic control.^5^ Beyond their metabolic efficacy, emerging real-world data suggest that GLP-1 therapy may be associated with reductions in cardiometabolic risk, psychiatric comorbidity, all-cause mortality, and the incidence of psoriatic arthritis among patients with psoriasis.^6^, ^7^, ^8^ Whether these agents exert direct disease-modifying effects on cutaneous inflammation, however, remains undefined.

The present study was designed to address this gap by evaluating the efficacy of GLP-1 receptor agonists in psoriasis.

## Methods

### Study design and population

We conducted a retrospective, single-center cohort study at the Division of Dermatology, Sheba Medical Center, Israel—the largest tertiary dermatology service in the country—between January 1, 2020, and June 30, 2025. Consecutive adult patients (≥18 years) with a dermatologist-confirmed diagnosis of psoriasis and comorbid obesity were screened for eligibility. Obesity was defined as a BMI ≥30 kg/m^2^, or ≥27 kg/m^2^ in the presence of obesity-related metabolic comorbidities, in accordance with national regulatory criteria for initiation of GLP-1 receptor agonist therapy in Israel. Clinical data were obtained from the institutional electronic health record system and verified by board-certified dermatologists.

Two cohorts were constructed. Cohort A included patients with psoriasis and obesity who were not receiving any systemic anti-psoriatic therapy, enabling isolation of the independent effect of GLP-1 receptor agonists on psoriasis outcomes. Cohort B included patients treated with systemic biologic therapy, comparing those who additionally initiated GLP-1 receptor agonists with biologic-treated controls who did not. For both cohorts, patients were excluded if they had received systemic immunomodulatory agents (methotrexate, cyclosporine, acitretin, apremilast, or other non-biologic systemic therapies) within the 12 weeks preceding the index date, to avoid overlapping therapeutic effects. Additional exclusion criteria included a history of inflammatory bowel disease, active malignancy, uncontrolled endocrinopathies, or incomplete follow-up documentation.

### Exposure

The exposure of interest was treatment with a glucagon-like peptide-1 (GLP-1) receptor agonist, including semaglutide, liraglutide, dulaglutide, exenatide, or tirzepatide. Patients were classified as exposed if they initiated a GLP-1 receptor agonist during the study period and maintained therapy for at least 12 consecutive weeks. Initiation of GLP-1 receptor agonist therapy was determined as part of routine clinical care, based on clinical indication, patient preference, and treating physician judgment, in accordance with national prescribing criteria. The index date was defined as the date of GLP-1 initiation.

The comparison group consisted of patients with psoriasis and obesity who were managed during the same period but did not receive GLP-1 therapy. Both groups received standard dermatologic and lifestyle care, including dietary counseling, physical activity recommendations, and topical-based management as clinically indicated.

### Data collection and variables

Demographic variables (age, sex, ethnicity), clinical characteristics (psoriasis duration, subtype, and severity), anthropometric measures (weight, BMI, waist circumference), and comorbidities (hypertension, diabetes mellitus, dyslipidemia, ischemic heart disease, nonalcoholic fatty liver disease, psychiatric disorders) were collected. Smoking status and medication history were recorded.

Psoriasis severity was assessed using the Investigator’s Global Assessment (IGA), with scores ranging from 0 (clear) to 4 (severe).^9^ Disease improvement was defined as achievement of an IGA 0/1 response (“clear” or “almost clear”) at week 48. Laboratory markers—including fasting glucose, HbA1c, lipid profile, liver function tests, and C-reactive protein—were extracted when available. Weight measurements were standardized across clinic visits and verified through the hospital’s electronic database.

### Outcomes

The primary outcomes were (1) the proportion of patients achieving an IGA score of 0 or 1 (clear or almost clear) at 48 weeks, as assessed by board-certified dermatologists in a blinded fashion, and (2) the correlation between weight reduction and improvement in cutaneous disease activity. All patients were evaluated at the 48-week time point.

### Statistical analysis

Continuous variables were expressed as mean (SD) or median (IQR), and categorical variables as counts (percentages). Group differences were assessed with *t* tests or χ^2^ tests, as appropriate. Missing outcome data (<10%) were handled using a nonresponder imputation approach, applying the last observation carried forward (LOCF) method. Logistic regression was used to estimate odds ratios (OR) and 95% confidence intervals (CI) for treatment response, adjusted for baseline covariates, including age, sex, BMI, smoking status, psoriasis duration, and comorbidities (diabetes mellitus, hypertension, dyslipidemia, ischemic heart disease). Kendall’s τ-b correlation for censored data was estimated using the *cenken* function from the NADA2 package to evaluate the association between the degree of weight reduction and the time to achievement of an IGA 0/1 response.

To further explore the mechanistic contribution of weight change to treatment response, causal mediation analyses were performed using the mediation package in R. The total effect of treatment on response was decomposed into the direct effect (treatment effect independent of weight change) and the indirect effect (mediated through weight reduction). Mediation models were based on logistic regression for the binary outcome (treatment response) and linear regression for the continuous mediator (change in weight), adjusting for the same baseline covariates as in the main models. The proportion of the total effect mediated by weight reduction was estimated with nonparametric bootstrapping (1000 iterations) to obtain bias-corrected 95% CIs. To account for multiple comparisons, *P* values were adjusted using the false discovery rate (FDR) method according to Benjamini and Hochberg, where applicable. Statistical significance was set at *P* < .05. Analyses were performed using R (version 4.3.2).

## Results

### Study population

Cohort A included 94 patients in total, comprising 36 individuals treated with GLP-1 analogs and 58 control subjects. Patients in the GLP-1 group were significantly older than controls (mean age, 65.1 ± 10.6 vs 54.6 ± 18.5 years; *P* < .001). The sex distribution did not differ significantly between groups (50% vs 69% male; *P* = .11). The prevalence of comorbid hypertension, and dyslipidemia was comparable between the GLP-1 and control groups (11.1% vs 3.4%, 11.1% vs 3.4%, and 38.9% vs 46.5%, respectively; all *P* > .05). Diabetes was more common in the GLP-1 group (22.2% vs 3.4%, *P* = .009) (Table I).

**Table I:** Study population

Characteristic	Cohort A: No systemic			Cohort B: Biologics		
	GLP-1 (*n* = 36)	Controls (*n* = 58)	*P* value	GLP-1 + Biologics (*n* = 20)	Biologics alone (*n* = 40)	*P* value
Age	65.1 (10.6)	54.6 (18.5)	**<.001**	58.3 (12.1)	56.7 (11.4)	.61
Sex, *n* (%)						.79
Male	18 (50)	40 (69)	.11	11 (55.0)	20 (50.0)	
Female	18 (50)	18 (31)		9 (45.0)	20 (50.0)	
BMI	33.5 (5.1)	33.3 (9.6)	.69	35.8 (6.4)	34.9 (6.0)	.57
Comorbidities, *n* (%)						
Diabetes	8 (22.2)	2 (3.4)	**.009**	6 (30.0)	8 (20.0)	.39
Hypertension	4 (11.1)	2 (3.4)	.3	7 (35.0)	11 (27.5)	.56
Dyslipidemia	14 (38.9)	27 (46.5)	.61	10 (50.0)	18 (45.0)	.79
GLP-1 receptor agonist, *n* (%)						
Semaglutide	28 (77.8)			15 (75.0)		
Liraglutide	4 (11.1)			3 (15.0)		
Dulaglutide	2 (5.6)			1 (5.0)		
Exenatide	1 (2.8)			0 (0)		
Tirzepatide	1 (2.8)			1 (5.0)		

Bolded *P* values indicate statistical significance.

In Cohort B, which included 60 biologic-treated patients, baseline characteristics were well balanced between those who initiated GLP-1 therapy and biologic-treated controls. Patients receiving combination GLP-1 plus biologic therapy had a similar mean age compared with biologics alone (58.3 ± 12.1 vs 56.7 ± 11.4 years; *P* = .61) and comparable BMI (35.8 ± 6.4 vs 34.9 ± 6.0 kg/m^2^; *P* = .57). Sex distribution did not differ significantly (55% vs 50% male; *P* = .79). The prevalence of diabetes, hypertension, and dyslipidemia was also similar across groups (30.0% vs 20.0%, 35.0% vs 27.5%, and 50.0% vs 45.0%, respectively; all *P* > .05).

### Efficacy outcomes

In cohort A, an IGA 0/1 response was achieved by 16 of 36 patients (44.4%) in the GLP-1 group compared with 7 of 58 patients (12.1%) in the control group (*P* < .001). IGA 0 response was observed in 10 of 36 patients (27.8%) in the GLP-1 group. In Cohort B, 17 of 20 patients (85.0%) receiving combination GLP-1 plus biologic therapy achieved an IGA 0/1 response compared with 21 of 40 patients (52.5%) treated with biologics alone, a statistically significant difference (*P* = .029). Overall, the addition of GLP-1 therapy increased the IGA 0/1 response rate by 32.5 percentage points.

**Fig 1:**
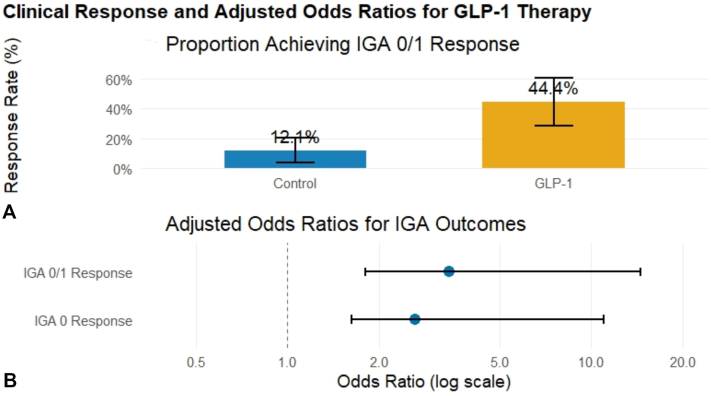
Clinical response and adjusted odds ratios for GLP-1 Analog therapy in psoriasis. **A,** Proportion of patients achieving an Investigator’s Global Assessment (IGA) 0/1 response at follow-up in the GLP-1 group compared with matched controls receiving topical therapy alone. Error bars represent 95% confidence intervals. **B,** Adjusted odds ratios (ORs) for achieving IGA 0/1 response and complete clearance (IGA 0) associated with GLP-1 analog therapy in logistic regression models adjusted for age, sex, and BMI using penalized estimation. The *dashed vertical line* indicates an OR of 1 (no effect).

In logistic regression models (both cohorts) adjusted for age, sex, and BMI (penalized to address quasi-separation), GLP-1 analog therapy remained associated with higher odds of achieving IGA 0/1 response (adjusted OR, 3.41; 95% CI, 1.80-14.52) and IGA 0 response (adjusted OR, 2.64; 95% CI, 1.63-10.98) (Fig.1). In analyses limited to patients receiving the most common GLP-1 analog (semaglutide), the association remained consistent, with higher odds of achieving IGA 0/1 (adjusted OR, 3.12; 95% CI, 1.42-13.07) and IGA 0 (adjusted OR, 2.41; 95% CI, 1.21-9.84).

To examine whether the improvement in psoriasis severity among GLP-1-treated patients was dependent on underlying metabolic status, outcomes were compared between diabetic and nondiabetic individuals. Among the 36 patients receiving GLP-1 analogs (cohort A), 8 (22.2%) had diabetes. An IGA 0/1 response was achieved by 4 of 8 patients with diabetics (50.0%) and 12 of 28 patients with nondiabetics (42.9%; *P* = .73).

### Correlation between weight reduction and cutaneous efficacy

**Fig 2:**
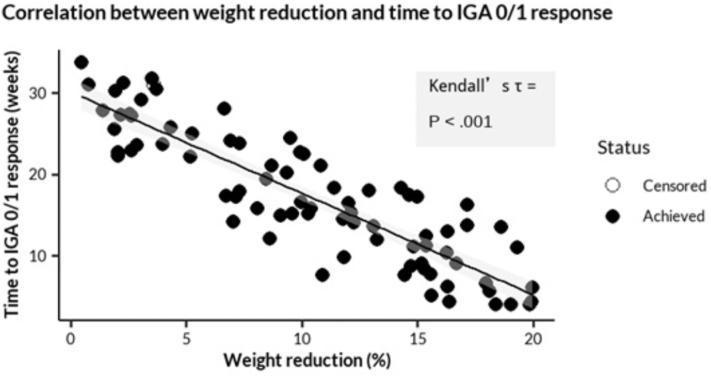
Correlation between weight loss and psoriasis improvement in the GLP-1 analog group.

In the GLP-1 analog group, greater weight reduction was strongly associated with a shorter time to achievement of an IGA 0/1 response in both cohorts, as shown by a significant Kendall’s τ-b correlation for censored data (τ = −0.59; *P* < .001) (Fig 2).

**Fig 3:**
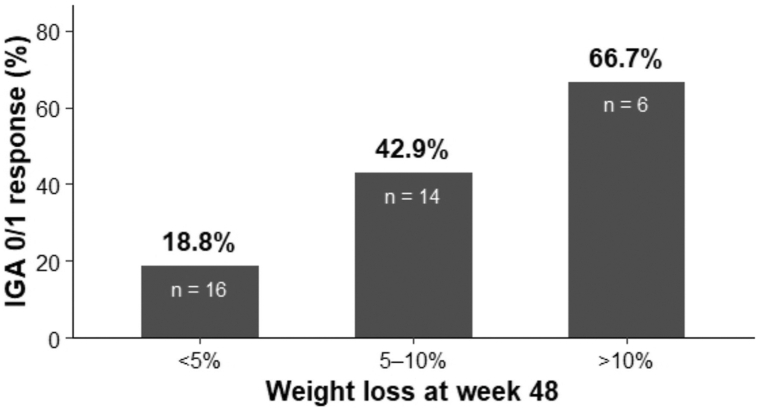
Dose-dependent relationship between weight loss and psoriasis improvement among GLP-1-treated patients.

To explore a potential dose–response relationship between weight reduction and improvement in psoriasis severity, GLP-1–treated patients (Cohort A) were stratified by the degree of weight loss at week 48 into 3 categories: <5% (*n* = 16), 5% to 10% (*n* = 14), and >10% (*n* = 6). The proportion of patients achieving an IGA 0/1 response increased from 18.8% (3/16) in the <5% group to 42.9% (6/14) in the 5% to 10% group and 66.7% (4/6) among those with >10% weight loss (*P* for trend = .0286) (Fig 3).

To assess whether associations between GLP-1 analog use and psoriasis outcomes persisted after accounting for weight loss, regression models were further adjusted for percentage weight change. After inclusion of this covariate, the association between GLP-1 analog use and achievement of an IGA 0/1 response remained statistically significant but became more modest (adjusted OR, 2.47; 95% CI, 1.01-6.05; *P* = .046)

In mediation analyses, both total and decomposed effects were estimated. The total effect of treatment on achieving an IGA 0/1 response was significant (β = 1.36; 95% CI, 1.02-1.70; *P* < .001). Weight reduction was strongly associated with treatment assignment (β = −0.24; 95% CI, −0.33 to −0.15; *P* < .001) and independently predicted greater likelihood of achieving an IGA 0/1 response (β = 0.35; 95% CI, 0.18-0.51; *P* < .001). The estimated indirect (mediated) effect through weight reduction accounted for approximately 38% (95% CI, 29% to 47%) of the total treatment effect, while the direct effect of treatment, independent of weight change, remained significant (*P* < .001). Bootstrap sensitivity analyses (1000 replications) yielded consistent results.

## Discussion

The association between psoriasis, obesity, and metabolic syndrome is well established; however, therapeutic strategies targeting this metabolic component have been infrequently explored in diet-focused studies. In a randomized clinical trial, adherence to a Mediterranean diet resulted in a significant improvement in psoriasis severity, accompanied by a reduction in glycated hemoglobin levels, underscoring the metabolic component of psoriasis pathogenesis.^10^ In another trial, dietary intervention resulted in a 48% improvement in mean Psoriasis Area and Severity Index (PASI) and a ≥50% reduction in PASI scores in 49.7% of participants.^11^ In an open-label study, dietary reduction of saturated free fatty acids was shown to improve psoriatic disease activity and systemic inflammatory markers independently of weight loss.^12^ Weight loss attained through bariatric surgery has also been shown to improve psoriasis severity.^13^^,^^14^ In general, weight loss appears to have a positive effect on psoriasis.^15^, ^16^, ^17^, ^18^ BMI is well known to correlate with psoriasis severity^19^; consequently, reductions in BMI may contribute to meaningful improvement in disease activity. In this study, we demonstrate the potential of GLP-1 analogs in improving psoriatic skin disease.

Psoriasis improvement with GLP-1 receptor agonists has been reported primarily in small studies of patients with concomitant type 2 diabetes. In a randomized controlled trial by Lin et al, liraglutide significantly reduced PASI and DLQI scores over 12 weeks compared with controls, accompanied by histologic improvement and decreased cutaneous expression of IL-17, IL-23, and TNF-α.^20^ In another randomized clinical study of 31 patients who have obesity with psoriasis and type 2 diabetes, semaglutide significantly reduced psoriasis severity, with median PASI scores improving from 21 to 10 and DLQI scores improving from 14 to 4. Treatment also led to meaningful reductions in systemic inflammatory markers, including IL-6 and CRP, as well as favorable metabolic effects such as decreases in BMI and LDL cholesterol.^21^ A prospective study of 7 patients similarly demonstrated substantial PASI reduction (mean 15.7 to 2.2) with parallel DLQI improvement, alongside favorable metabolic changes—including reductions in BMI, waist circumference, HbA1c, and HOMA-IR—and decreased epidermal thickness on biopsy.^22^ A separate before–after study by Marietta et al further supported an immune-metabolic mechanism: liraglutide improved PASI and DLQI scores over 10 weeks, reduced body weight, and increased circulating invariant natural killer T cells with a trend toward lower monocyte TNF-α production.^23^ In contrast, a randomized placebo-controlled trial in patients who are glucose-tolerant with plaque psoriasis found no significant differences between liraglutide and placebo in PASI, DLQI, or hsCRP after 8 weeks, despite meaningful weight loss and reductions in cholesterol.^24^ Finally, several anecdotal case reports describe marked improvement in psoriasis with GLP-1 agonists,^25^, ^26^, ^27^ supporting a potential therapeutic signal but underscoring the need for adequately powered randomized studies in nondiabetic populations.

Compared with the prior literature—largely composed of small, short-duration randomized trials, metabolic intervention studies in patients with type 2 diabetes, and isolated case reports—our study offers several important strengths. First, it includes a substantially larger sample size than any previously published cohort evaluating GLP-1 receptor agonists for psoriasis, thereby providing more precise estimates of treatment effect and increasing generalizability beyond highly selected diabetic populations. Second, our design incorporates 2 distinct cohorts, including a biologic-treated subgroup, allowing us to examine not only the independent effect of GLP-1 therapy but also its *synergistic benefit when combined with biologic agents*. This synergistic signal—demonstrated by markedly higher IGA 0/1 response rates with combination therapy than with biologics alone—has not been evaluated in prior studies and provides novel evidence supporting an immune–metabolic interaction relevant to modern psoriasis therapeutics. Together, these features position our study as the largest and most clinically informative analysis to date of GLP-1 receptor agonists in psoriasis, expanding current understanding beyond diabetic cohorts and offering the first real-world insight into their additive effect alongside biologic therapy.

Limitations of this study include its retrospective design, which may introduce selection and information bias, as well as the relatively limited sample size, which constrains statistical power and the generalizability of findings. Accordingly, some estimates were accompanied by wide confidence intervals, reflecting residual uncertainty in effect size despite consistent directional associations across analyses. Second, the study population was restricted to patients with obesity, which limits the generalizability of the findings to nonobese individuals with psoriasis. Furthermore, the absence of longitudinal data on lifestyle factors, adherence, and concomitant therapies limits causal inference regarding treatment effects. The reliance on the *IGA* rather than the *Psoriasis Area and Severity Index* (PASI), owing to the retrospective nature of data collection, may also limit precision in quantifying disease severity. Lastly, the modest cohort size precluded meaningful subgroup analyses based on sex, baseline BMI, or psoriasis phenotype.

In summary, this study introduces GLP-1 analogs as a novel therapeutic target in psoriasis, highlighting the potential of metabolic modulation as an adjunctive approach to traditional immunomodulatory therapy. Future prospective, controlled studies integrating metabolic biomarkers, imaging, and immunophenotyping are warranted to define their role within the evolving therapeutic landscape of psoriasis.

## Conflicts of interest

None disclosed.

## References

[1] Armstrong A.W., Read C. (2020). Pathophysiology, clinical presentation, and treatment of psoriasis: a review. JAMA.

[2] Jensen P., Skov L. (2016). Psoriasis and obesity. Dermatology.

[3] Yanaba K., Umezawa Y., Ito T. (2014). Impact of obesity on the efficacy of ustekinumab in Japanese patients with psoriasis: a retrospective cohort study of 111 patients. Arch Dermatol Res.

[4] Pinter A., Gerdes S., Papavassilis C., Reinhardt M. (2020). Characterization of responder groups to secukinumab treatment in moderate to severe plaque psoriasis. J Dermatolog Treat.

[5] Popoviciu M.S., Păduraru L., Yahya G., Metwally K., Cavalu S. (2023). Emerging role of GLP-1 agonists in obesity: a comprehensive review of randomised controlled trials. Int J Mol Sci.

[6] Olbrich H., Kridin K., Zirpel H. (2025). GLP-1RA and reduced mortality, cardiovascular and psychiatric risks in psoriasis: a large-scale cohort study. Br J Dermatol.

[7] Sontam T., Chen H., Raman J., Chen A., Cho S.W., Tarbox M. (2025). Glucagon-like peptide-1 receptor agonist use in patients with psoriasis is associated with lower incidence of atherosclerotic cardiovascular disease: a retrospective cohort study using TriNetX. J Am Acad Dermatol.

[8] Hsiao Y.W., Huang Y.C. (2026). Glucagon-like peptide-1 receptor agonist therapy in patients with psoriasis is associated with decreased risk of psoriatic arthritis: a retrospective cohort study from the TriNetX database. J Am Acad Dermatol.

[9] Langley R.G., Feldman S.R., Nyirady J., van de Kerkhof P., Papavassilis C. (2015). The 5-point Investigator's Global Assessment (IGA) scale: a modified tool for evaluating plaque psoriasis severity in clinical trials. J Dermatolog Treat.

[10] Perez-Bootello J., Berna-Rico E., Abbad-Jaime de Aragon C. (2025). Mediterranean diet and patients with psoriasis: the MEDIPSO randomized clinical trial. JAMA Dermatol.

[11] Naldi L., Conti A., Cazzaniga S., Psoriasis Emilia Romagna Study Group (2014). Diet and physical exercise in psoriasis: a randomized controlled trial. Br J Dermatol.

[12] Saalbach A., Seitz A.T., Kohlmann J. (2023). Modulation of dietary fatty acids in an open-label study improves psoriasis and dampens the inflammatory activation status. Nutrients.

[13] Laskowski M., Schiöler L., Ottosson J. (2021). Impact of bariatric surgery on moderate to severe psoriasis: a retrospective nationwide registry study. Acta Derm Venereol.

[14] Hosseininasab A., Mosavari H., Rostami A. (2024). The long-term impact of bariatric surgery on psoriasis symptoms and severity: a prospective observational study. Surg Obes Relat Dis.

[15] Alotaibi H.A. (2018). Effects of weight loss on psoriasis: a review of clinical trials. Cureus.

[16] Debbaneh M., Millsop J.W., Bhatia B.K., Koo J., Liao W. (2014). Diet and psoriasis, part I: impact of weight loss interventions. J Am Acad Dermatol.

[17] Jensen P., Zachariae C., Christensen R. (2013). Effect of weight loss on the severity of psoriasis: a randomized clinical study. JAMA Dermatol.

[18] Jensen P., Christensen R., Zachariae C. (2016). Long-term effects of weight reduction on the severity of psoriasis in a cohort derived from a randomized trial: a prospective observational follow-up study. Am J Clin Nutr.

[19] Sobhan M., Farshchian M. (2017). Associations between body mass index and severity of psoriasis. Clin Cosmet Investig Dermatol.

[20] Lin L., Xu X., Yu Y. (2022). Glucagon-like peptide-1 receptor agonist liraglutide therapy for psoriasis patients with type 2 diabetes: a randomized-controlled trial. J Dermatolog Treat.

[21] Petković-Dabić J., Binić I., Carić B. (2025). Effects of semaglutide treatment on psoriatic lesions in obese patients with type 2 diabetes mellitus: an open-label, randomized clinical trial. Biomolecules.

[22] Xu X., Lin L., Chen P. (2019). Treatment with liraglutide, a glucagon-like peptide-1 analogue, improves effectively the skin lesions of psoriasis patients with type 2 diabetes: a prospective cohort study. Diabetes Res Clin Pract.

[23] Buysschaert M., Baeck M., Preumont V. (2014). Improvement of psoriasis during glucagon-like peptide-1 analogue therapy in type 2 diabetes is associated with decreasing dermal γδ T-cell number: a prospective case-series study. Br J Dermatol.

[24] Faurschou A., Gyldenløve M., Rohde U. (2015). Lack of effect of the glucagon-like peptide-1 receptor agonist liraglutide on psoriasis in glucose-tolerant patients--a randomized placebo-controlled trial. J Eur Acad Dermatol Venereol.

[25] Costanzo G., Curatolo S., Busà B., Belfiore A., Gullo D. (2021). Two birds one stone: semaglutide is highly effective against severe psoriasis in a type 2 diabetic patient. Endocrinol Diabetes Metab Case Rep.

[26] Faurschou A., Knop F.K., Thyssen J.P., Zachariae C., Skov L., Vilsbøll T. (2014). Improvement in psoriasis after treatment with the glucagon-like peptide-1 receptor agonist liraglutide. Acta Diabetol.

[27] Haran K., Johnson C.E., Smith P. (2024). Impact of GLP-1 receptor agonists on psoriasis and cardiovascular comorbidities: a narrative review. Psoriasis (Auckl).

